# Chemical Studies of Yellow Tamarillo (*Solanum betaceum* Cav.) Fruit Flavor by Using a Molecular Sensory Approach

**DOI:** 10.3390/molecules21121729

**Published:** 2016-12-16

**Authors:** Juliana María García, Laura Juliana Prieto, Alirio Guevara, Diana Malagon, Coralia Osorio

**Affiliations:** 1 Departamento de Química, Universidad Nacional de Colombia, AA 14490 Bogotá, Colombia; jumgarciach@unal.edu.co (J.M.G.); ljprietop@unal.edu.co (L.J.P.); 2 Disaromas S.A., Cra 46 No. 20A-90 Bogotá, Colombia; aliriog@disaromas.com (A.G.); malagondianam@gmail.com (D.M.)

**Keywords:** tree tomato, AEDA, rosmarinic acid, bitterness, taste, aroma

## Abstract

The odor-active volatile compounds of yellow tamarillo fruit (*S. betaceum* Cav.) were identified and quantified by using a sensomics approach, combining a gentle volatile extraction (solvent-assisted flavor evaporation (SAFE)), gas chromatography-mass spectrometry (GC-MS), and sensory analyses (gas chromatography-olfactometry (GC-O) and aroma extract dilution analysis (AEDA)). The medium-term purpose of this work is to evaluate the change of odor-active volatiles during processing. Thus, (*Z*)-3-hexenal, hexanal, and ethyl butanoate were identified as key aroma compounds of yellow tamarillo. The C_6_-aliphatic compounds, aliphatic esters, and terpenols were characterized as the volatiles responsible for the herbal-green, fruity, and fresh-mint odor notes of this variety, respectively. Additionally, one non-volatile compound contributing to the residual bitter taste of this fruit was isolated by a bioguided (taste sensory analyses) fractionation. The freeze-dried fruit was sequentially liquid-liquid partitioned with solvents of different polarity, and then the ethyl acetate fraction was submitted to size exclusion chromatography. Then, its structure was elucidated as rosmarinic acid, by using common spectroscopic methods (mass spectrometry (MS) and nuclear magnetic resonance (NMR)). The amount of rosmarinic acid was quantified as 46.17 ± 1.20 mg/100 g of dried fruit, by the external standard method. Its bitter taste threshold value was determined by using the 3AFC (alternative forced choice) method to be 37.00 ± 1.25 mg/L.

## 1. Introduction

The research area “sensomics” has been developed within the last ten years to identify, quantify, catalogue, and evaluate the sensory activity of volatile compounds that impart the typical flavor of food products. This molecular sensory approach combines analytical sensory evaluation tools involving trained human subjects with modern instrumental techniques (chromatography-mass spectrometry (GC-MS) or high-performance liquid chromatography-mass spectrometry (HPLC-MS), for volatile and non-volatile compounds, respectively), enabling the identification of the most important aroma and taste compounds from a food. Regarding volatile compounds, only those with the highest odor activity values (the ratio of the concentration and its threshold concentration) could be considered as key aroma compounds [1,2]. Whereas the volatiles inducing the exotic aroma of tropical fruits have been thoroughly investigated in recent years, the non-volatile taste-active molecules in those fruits is an unexplored field. The knowledge of the chemical structures and sensory properties of key taste molecules would be helpful for the development of added-value food products. Additionally, the odor-taste interactions have been the target of recent studies. For example, Suess et al. [3] reported that the odor-active citronellal, to significantly decrease the perceived bitterness of a black tea infusion, as well as caffeine solutions. Cell-based functional experiments, revealed (*R*)-citronellal to completely block caffeine-induced calcium signals in TAS2R43-expressing cells.

**Figure 1:**
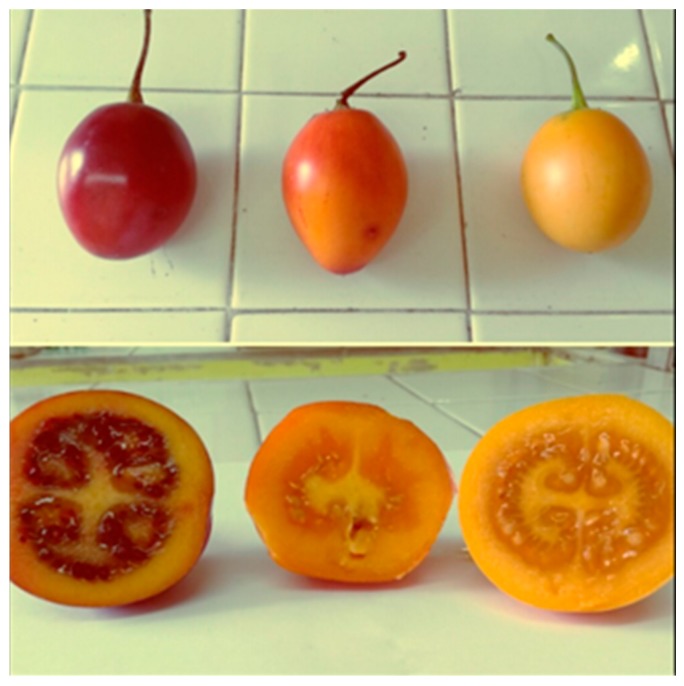
Commercial varieties of tamarillo, red; orange; and yellow-fleshed fruits.

*Solanum betaceum* is a shrub native to the Andes, specifically in Peru, Ecuador, and Colombia, that belongs to the Solanaceae family. The fruits are oval, covered by a thick, smooth, and shiny peel, with a red, orange, or yellow flesh, depending on the variety (Figure 1). Inside, its texture is firm, and juicy, with a bitter-sweet taste. In the center of the fruit, there are a large number of flat seeds surrounded by a smooth pulp. This fruit is source of vitamins A, B_6_, C, and E, and is also rich in calcium, iron, and phosphorus. Yellow fruits are considered promising because of their intense flavor, but some characteristic residual bitter and astringent flavors are undesirable by the consumers.

Some scientific studies related to the physicochemical composition, volatiles, pigment (carotenoids and anthocyanins), and polyphenol contents of this fruit have been published [4,5,6,7]. The volatile studies on red-variety fruits showed that the major constituents of pentane-dichloromethane liquid-liquid extract were methyl hexanoate, (*E*)-hex-2-enal, (*Z*)-hex-3-en-1-ol, eugenol, and 4-allyl-2,6-dimethoxyphenol [8]. Durant et al. [9] recently characterized the volatile compounds of two varieties of *S. betaceum* by means of headspace solid-phase microextraction (HS-SPME) coupled with gas chromatography-mass spectrometry (GC-MS). They reported that golden-yellow cultivars contained higher levels of esters and terpenes, with α-terpineol, methyl hexanoate, ethyl octanoate, ethyl hexanoate, and 1,8-cineole being the major volatile compounds detected in this variety. However, despite those studies, so far no systematic study aiming at the identification of the odor-active volatiles in yellow tamarillo has been performed before.

Thus, as part of our current studies on the bioprospecting of tropical fruits [10], the aim of the present work was to identify and quantify the odor-active volatile compounds in yellow tamarillo fruit pulp, as well as non-volatile compounds contributing to the bitter residual taste, by using the molecular sensory approach.

## 2. Results and Discussion

### 2.1. Odor-Active Volatiles in Yellow Tamarillo Fruit

**Figure 2:**
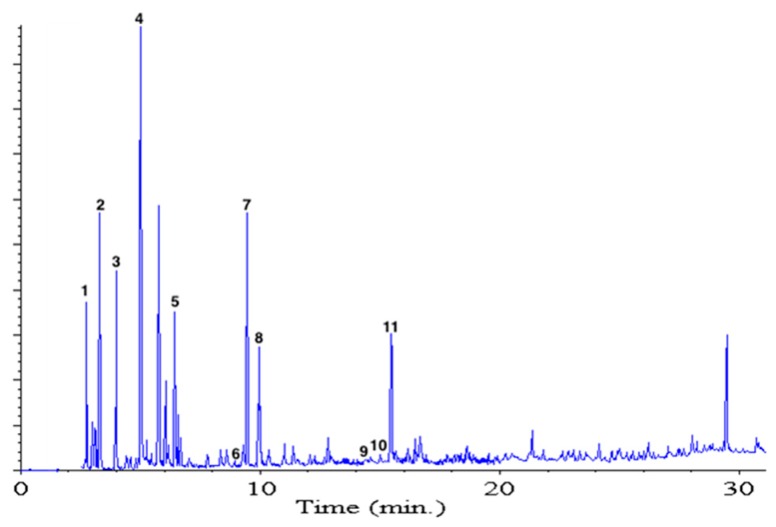
GC analyses on an FFAP column of the volatile compounds from yellow tamarillo fruit obtained by SAFE distillation. Numbers correspond to those in Table 1.

The *S. betaceum* fruits were homogenized with sodium sulfate to reduce the amount of water and improve the extraction efficiency, and after that, the resemblance of fruit aroma was checked in the organic extract before solvent-assisted flavor evaporation (SAFE) extraction. Chromatographic analysis of SAFE extract is showed in Figure 2. From the mixture of volatile compounds, only eleven odor-active volatile compounds were revealed after gas chromatography-olfactometry (GC-O) (aroma extract dilution analysis (AEDA)). In Table 1, these compounds are listed in elution order in the polar FFAP column, their odor description is reported, as well as the results of their quantitation. The odorants with higher FD values were (*Z*)-3-hexenal, ethyl butanoate, 1,8-cineole, terpinen-4-ol, and 2,3-butanediol. Ethyl butanoate, 1,8-cineole, and terpinen-4-ol had been also reported by Durant et al. [9] in yellow tamarillo from Panamá. However, hexanal, (*Z*)-3-hexenal, 4-hydroxy-4-methyl-2-pentanone, (*Z*)-3-hexenol, and 2,3-butanediol, among others, were not detected under the method used by them (headspace solid phase microextraction (HS-SPME)).

**Figure 3:**
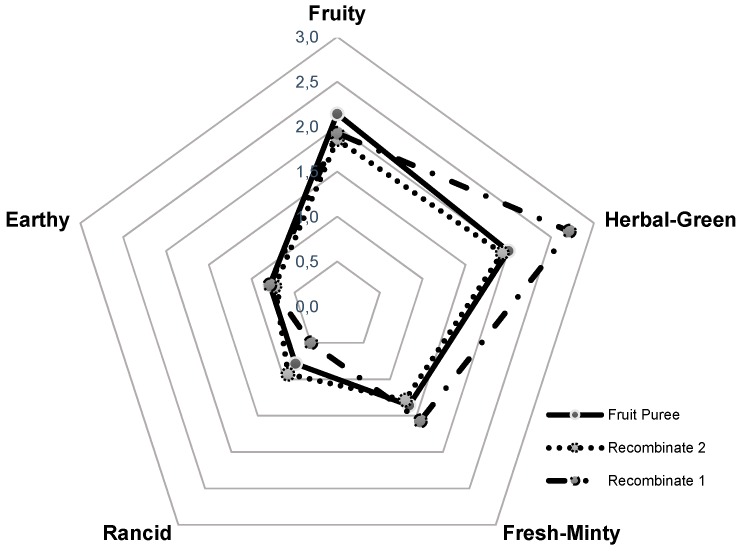
Aroma sensory profile comparison of yellow tamarillo fruit and recombinated mixtures.

Based on quantitative data and the odor threshold reported in literature [11], the odor activity values (OAVs) were calculated showing the relevance of each compound in the overall flavor of the fruit and finding that only nine volatile compounds exhibited OAVs higher than one. From these data, (*Z*)-3-hexenal, hexanal and ethyl butanoate were found as key aroma compounds in yellow tamarillo. Methyl butanoate, 1,8-cineole, and (*Z*)-3-hexenol were also significant; in contrast, the OAVs of hexanol and 2,3-butanediol showed that they are not so relevant for the aroma of this fruit. The results obtained by GC-O are in agreement with the sensory analyses performed by a trained panel on the fruit puree (Figure 3), where the most significant odor notes were herbal-green, attributable to C_6_-aliphatic compounds; fruity, from esters; and fresh-minty, from some terpenols.

Recombination studies were developed to validate the identification and quantitation processes. Therefore, an aqueous solution containing the nine odorants with OAVs greater that one (recombinate 1), was sensory compared with the tamarillo fruit puree. However, the herbal-green note due to (*Z*)-3-hexenal was over-expressed (Figure 3). It has been reported in the literature [12] that this compound is formed upon plant tissue disruption from linolenic acid, and the homogenization of the fruit before workup is the factor that increases the original (*Z*)-3-hexenal concentration and, therefore, produces a high intensity of the green odor note. For this reason, a second recombinate was prepared reducing the amount of this compound as it is indicated in the material and method section. The sensory panel concluded that this aroma resembled more the aroma of the fresh fruit, and the green odor-note was modulated.

### 2.2. Bitter-Active Compounds in Yellow Tamarillo Fruit

A polar extract (acetone-water) was obtained from lyophilized fruit. In order to obtain as much of the taste-active compounds as possible, a successive partition with solvents of different polarity was performed. Five fractions were obtained (F.Ether, F.DCM, F.EtOAc, F.BuOH, and F.Aqueous) in the first step of fractionation, and they were evaluated by the sensory panel to define the most relevant taste attributes. The five fractions were evaluated by a trained panel, showing that the taste descriptors for this fruit are: salty, bitter, sour, umami, astringent, and the characteristic tamarillo-like.

**Figure 4:**
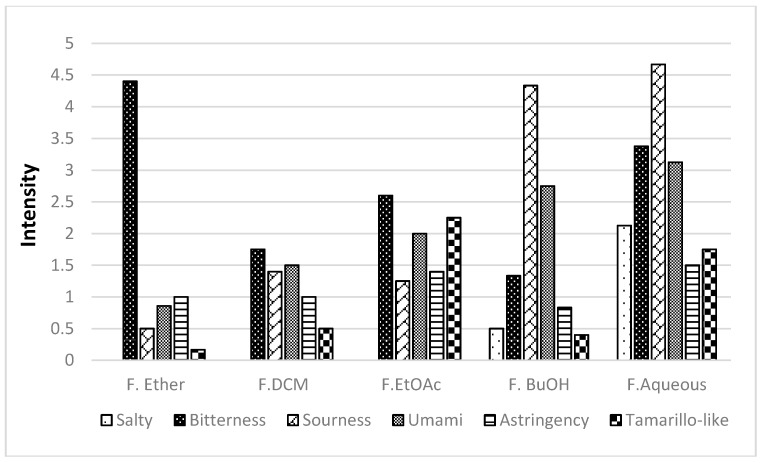
Taste intensity profile of the five yellow tamarillo fractions.

Figure 4 shows the distribution of taste descriptors in each fraction. This analysis revealed that it is not possible to differentiate each fraction based on their taste. Noticeably, the most polar fractions, F.Aqueous and F.BuOH, seemed to be related, with an intense contribution of sour and umami taste descriptors. Tamarillo-like taste note was found mainly in F.EtOAc and F.Aqueous. The bitter taste was chosen to bioguide the further fractionation, because it is undesirable for some consumers and the reason for their rejection to tamarillo juice or processed products.

Thus, F.EtOAC was selected because it exhibited not only bitter, but also the characteristic tamarillo-like and umami descriptors, and also because the sourness was low in comparison with the other fractions. It was subsequently fractionated by size exclusion chromatography to obtain four fractions (F1–F4), which also were evaluated by the sensory panel. From F2, compound **1** was isolated as a bitter contributor in yellow tamarillo, and its purity was confirmed by using the universal HPLC detectors: MS and ELSD (Evaporative Light Scattering Detector).

**Figure 5:**
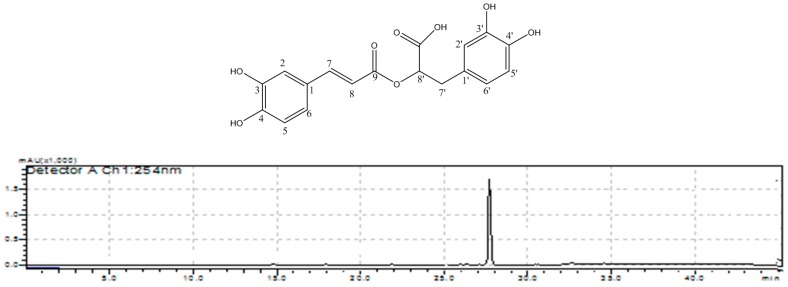
HPLC-UV-VIS analysis of F_2_ containing pure compound **1** (rosmarinic acid) (*λ* = 340 nm, Poreshell 120 SB-C_18_ column).

The electrospray ionization (ESI)-MS spectrum in negative mode of compound **1** with retention time of 27.8 min showed a pseudomolecular ion *m*/*z* 359 [M − H]^−^. In positive mode the following ion fragments were detected: an adduct ion at *m*/*z* 399 [M + K]^+^, the pseudomolecular ion at *m*/*z* 361 [M + H]^+^, as well as the ion at *m*/*z* 343 corresponding to the loss of water from the protonated ion [M − H_2_O + H]^+^. With these results a molecular mass of 360 was confirmed for compound **1**. The NMR data showed the presence of aromatic compound with a *trans* (*J*_7–8_ = 15.9 Hz) double bound conjugated with a carbonyl group. The analysis of these data allow to conclude that compound **1** was the rosmarinic acid (Figure 5), because the NMR data were in good agreement with those published elsewhere [13]. This compound was reported as constituent of tamarillo from Ecuador based only on HPLC-MS analysis [7]; however, this is the first time that rosmarinic acid is characterized as a bitter compound. This phenolic acid is known to have many biological activities, including hepatoprotective activities in liver diseases [14].

Additionally, the bitter taste threshold value for rosmarinic acid was determined for the first time using the 3AFC (alternative forced choice) method as 37.00 ± 1.25 mg/L, because it was not reported before in the literature. The amount of this compound in yellow tamarillo accounted for 46.17 ± 1.20 mg/100 g of dried fruit, a higher value than what the threshold would explain as the bitter residual taste that is perceived in this fruit.

Here, the identification of one bitter compound in yellow tamarillo is reported; the other bitter fractions (see Figure 4) of this fruit should be checked to identify other non-volatile compounds contributing to this undesirable flavor note.

## 3. Materials and Methods

### 3.1. General

^1^H- and ^13^C-NMR (400 and 100 MHz, respectively) spectra were acquired on a Bruker DRX400 spectrometer (Bruker, Karlsruhe, Germany). Data processing was performed using MestReNova 6.2 (Mestrelab Research SL, San Diego, CA, USA). NMR spectra were recorded in DMSO-*d*_6_ and referenced to the residual non-deuterated solvent signal at δ_H_ 2.50 ppm. GC-FID and GC-O analyses were performed by using an HP 5890 gas chromatograph (Hewlett-Packard, San Diego, CA, USA). GC-MS (EIMS, 70 eV) analyses were carried out on a GC Agilent 7890B gas chromatograph equipped with a 5977A MSD mass selective detector (Agilent Technologies Inc., Wilmington, DE, USA). MS data were recorded between 40–400 u, and processed by Mass Hunter software. Wiley library 10ª edition with MS NIST 2011 (Ringoes, NJ, USA) was used to help in the compound identification. The analytical UHPLC-PDA-ELSD was performed on a Thermo Scientific Dionex UltiMate 3000 (Donierstr, Germany) system equipped with an autosampler, quaternary pump, PDA (Photodiode Array Detector) detector and ELSD detector (SEDEX, Alfortville Cedex, France). HPLC-ESI/MS was performed in a Shimadzu LCMS-2010 system (Shimadzu, Tokyo, Japan) equipped with a UV-VIS detector (SPD-10A) and two pumps (LC-10AD) coupled in-line with a MS-2010 mass spectrometer. The equipment also included an on-line DGU-14A degasser and a Rheodyne injection valve with a 5 μL loop.

### 3.2. Fruits

Fresh yellow tamarillo (*Solanum betaceum* Cav.) fruits were obtained from a local orchard in Bogotá (Colombia) and processed immediately upon arriving at the laboratory. Their ripening stage was selected according to their peel color (100% yellow), pH value of fruit pulp was 3.87 ± 0.03, and soluble solids content (SS) was 10.05 ± 0.23 °Brix (data are given as average ± standard deviation *n* = 3).

### 3.3. Chemicals

Dichloromethane, acetone, methanol, diethyl ether, ethyl acetate, *n*-butanol, sodium sulphate (anhydrous), and *n*-alkane mix (C_8_–C_26_) were acquired from Merck (Darmstadt, Germany). Methanol and acetonitrile were HPLC-MS grade also from Merck (Darmstadt, Germany) for HPLC-MS analyses. Dichloromethane was distilled prior to use during volatile analysis. Pure reference standards of methyl butanoate, hexanal, (*Z*)-3-hexenal, 1,8-cineole, hexanol, (*Z*)-3-hexen-1-ol, and ethyl 3-hydroxy-butanoate were purchased from Sigma-Aldrich (Milwaukee, WI, USA). Pure reference standards of 4-hydroxy-4-methyl-2-pentanone, ethyl butanoate, 2,3-butanediol, and terpinen-4-ol were generously supplied by Disaromas S.A. (Bogotá, Colombia).

### 3.4. Isolation of Yellow Tamarillo Volatile Extract

Fruit pulp (492 g) was homogenized using a commercial stainless steel blender. Dichloromethane (100 mL) was added to the puree, and the mixture was cooled in an ice bath. With continuous stirring and cooling, anhydrous sodium sulfate (50 g) was added in small portions. The so-obtained extract was filtered through defatted cotton wool and the sodium sulfate/fruit powder obtained was washed with another portion of dichloromethane (200 mL). The combined organic phases, exhibiting the characteristic aroma of yellow *S. betaceum* fruits were extracted with the SAFE technique [15]. Then, the organic fraction was dried over anhydrous sodium sulfate, filtered, and concentrated to 1 mL using a Vigreux column (50 cm × 1 cm) at 37 °C.

### 3.5. Gas Chromatography–FID and Gas Chromatography Olfactometry (GC-O)

Two capillary columns DB-FFAP and HP-5 (each 30 m × 0.32 mm i.d., 0.25 μm film thickness; J and W Scientific, Chromatographie-Handel Müller, Fridolfing, Germany, and Restek, Bellefonte, PA, USA, respectively) were used. The samples were injected in a split/splitless injection port at 230 °C in split mode (1:10). The column oven was programmed from 40 °C (after 2 min) to 240 °C at 4 °C/min and finally held at 240 °C and 300 °C for DB-5 for 10 min. Helium (2.0 mL/min) was used as the carrier. For GC-O analyses, the end of the capillary was connected to a deactivated Y-shaped glass splitter (Chromatographie Handel Mueller, Fridolfing, Germany) dividing the effluent into two equal parts, one for FID (230 °C) and the other for heated sniffing port (200 °C) by using deactivated fused silica capillaries of the same length. Chromatographic conditions in GCMS analyses were the same as those above-mentioned for GC-FID analyses.

### 3.6. Aroma Extract Dilution Analysis (AEDA)

The SAFE extract was stepwise diluted to obtain dilutions of 2^n^, and each solution was analyzed by GC-O in splitless mode, using a capillary FFAP column under the above-described conditions. The odor activity of each compound, expressed as the flavor dilution (FD) factor, was determined as the greatest dilution at which that compound was still detected by comparing all of the runs [16,17].

### 3.7. Volatile Compound Identification and Quantitation

Linear retention indexes (LRI) of the odor-active compounds were calculated by using a mixture of alkanes (C_7_–C_26_) as external references. The identification of volatile compounds was completed by comparison of their retention indexes, mass spectra, and odor notes with those exhibited by standard solutions of volatile compounds in dichloromethane (50 µg/mL).

Quantitative analyses of yellow tamarillo odor-active volatiles exhibiting dilution factors higher than eight were done by the internal standard (IS) method. For this purpose, hexyl acetate (Sigma Chem. Co., St. Louis, MO, USA) was dissolved in the extraction solvent (100 µg/mL), and added to the yellow tamarillo solution as the internal standard at the beginning of extraction. To determine the response factor for each volatile compound, calibration curves were constructed using a series of solutions of varying nominal concentrations containing each analyte (IS:analyte from 1:5 to 5:1), where the slope was assumed as the response factor. An identical amount of the internal standard was added to each solution and the corresponding chromatograms obtained [18]. All data were obtained in triplicate. The concentration of each analyte was calculated by comparison of GC-FID signals with those of standards, taking into account the relative response factor, according to the following equation:

$$\left[\;\right]x\;=\;\frac{A_{X}}{A_{\mathrm{istd}}}*\;\frac{µg\;\mathrm{IS}}{\mathrm{kg}\;\mathrm{fruit}}{*\;\mathrm{RF}}^{-1}$$

where, []x is the analyte concentration in mg/kg fruit, AX is the analyte area, A_istd_ is the internal standard area, and RF is the response factor. Key aroma compounds were determined based on their OAV (odor activity value = concentration divided by odor threshold in water from the literature [11]).

### 3.8. Isolation and Identification of Bitter Taste Active Compounds

Yellow tamarillo fruits (4.8 kg) were peeled, cut into slices and lyophilized to obtain 676 g of dried fruit. The procedure of fractionation reported by Isaza et al. [19] was followed. Thus, portions of 90 g (total of 676 g) of dried fruit were homogenized in 70% aqueous acetone (300 mL × 3), filtered, and concentrated in vacuum. The concentrated solution was extracted successively with ethyl ether, dichloromethane, ethyl acetate, and *n*-butanol to yield respective F.Ether (3.2 g), F.DCM (2.2 g), F.EtOAc (1.1 g), and F.BuOH (6.3 g) fractions, and the aqueous residue, F.Aqueous (93.6 g). The EtOAc extract (68 mg) was fractionated on a Toyopearl HW-40S column (100 mg, Tosoh Bioscience, Tokyo, Japan) to give four fractions: 40% MeOH in H_2_O (17 mg), 70% MeOH in H_2_O (23 mg), (CH_3_)_2_CO–MeOH–H_2_O (2:5:3, *v*/*v*) (8 mg), and (CH_3_)_2_CO–H_2_O (7:3, *v*/*v*) (4 mg). The 70% MeOH afforded 23 mg of compound **1**, which was described as bitter after sensory analyses.

Rosmarinic acid (compound **1**) was obtained as a white solid. ESI-MS *m*/*z* (negative mode): 359 [M − H]^−^ {100}; (positive mode): 399 [M + K]^+^ {5}, 361 [M + H]^+^ {10}, 343 [M − H_2_O + H]^+^ {15}, 163 [C_9_H_7_O_3_]^+^ {60}. ^1^H-NMR (400 MHz, *d*_6_-DMSO): δ 2.89 (1 H, d, *J* = 14.0 Hz, H-7b′), 2.98 (1 H, dd, *J* = 14.0, 4.0 Hz, H-7a′), 5.02 (1 H, m, H-8′), 6.24 (1 H, d, *J* = 15.9 Hz, H-8), 6.53 (1 H, d, *J* = 8.0 Hz, H-5′), 6.64 (1 H, d, *J* = 8.0 Hz, H-6′), 6.69 (1 H, brs, H-2′), 6.77 (1 H, d, *J* = 8.1 Hz, H-5), 7.00 (1 H, d, *J* = 8.2 Hz, H-6), 7.06 (1 H, d, *J* = 1.8 Hz, H-2), 7.46 (1 H, d, *J* = 15.9 Hz, H-7); ^13^C-NMR (100 MHz, *d*_6_-DMSO): δ 36.7 (C-7′), 73.7 (C-8′), 113.9 (C-8), 115.3 (C-2), 115.9 (C-6′), 116.3 (C-5), 117.1 (C-2′), 120.5 (C-5′), 122.0 (C-6), 125.8 (C-1), 128.1 (C-1′), 144.3 (C-3′), 145.4 (C-4′), 146.1 (C-7), 146.2 (C-4), 149.1 (C-3), 166.7 (C-9), 171.6 (C-9′).

### 3.9. LC-ESI/MS Analyses

The fractions of tamarillo fruit and compound **1** were analyzed by HPLC-ESI/MS. UV, and MS data were acquired and processed using Shimadzu LCMS Solution software (ver. 5.6, Kyoto, Japan). A Poroshell 120 SB-C_18_ 2.7 μm column (150 × 4.6 mm i.d., Agilent, USA) was used. The solvent system was a linear gradient of acetonitrile: water (formic acid 0.1%, *v*/*v*), as follows: from 0–2 min, acetonitrile 3%, from 2–30 min, 25%, from 30–40 min, 85%, from 40–45 min, 25%, and then to initial conditions at the flow rate of 0.8 mL/min. The electrospray ionization (ESI) probe was operated in positive and negative scan mode: CDL, 250 °C; block at 240 °C; flow gas (N_2_) at 1.5 L/min; CDL voltage, 150.0 kV; Q array voltage RF 150 V; detector voltage, 1.8 kV; and scan range *m*/*z* 100–800. Prior to injection (volume of 100 μL), all samples were filtered through a 0.45 μm Millipore membrane filter.

### 3.10. Quantitation of Rosmarinic Acid (Compound ***1***)

The rosmarinic acid was quantitated by using the purified compound as external standard in the UHPLC-PDA-ELSD equipment. Five solutions of rosmarinic acid (7, 40, 100, 150, and 400 ppm) were prepared in acetonitrile–water (4:1, *v*/*v*). The standards solutions and the sample were analyzed at λ 340 nm in the same chromatographic conditions used for LCMS analyses, and these data were used to establish the regression equation (*y* = 0.0236*x* + 0.006) that allow the quantitation of the rosmarinic acid. This graph showed good linearity with the regression coefficient, ranging from 0.98 to 0.99. All samples were analyzed in triplicate, and the mean ± standard deviation was reported.

### 3.11. Sensory Analyses

*Aroma*. The aroma profile of fruit pulp was evaluated by seven trained panelists, from the Departamento de Química, Universidad Nacional de Colombia. Homogenized fruit pulp (10 g) was placed in glass vessels which were closed with ground glass lids at 20 ± 1 °C. The assessors were asked to orthonasally evaluate the intensity of five descriptive sensory attributes from the overall aroma of the yellow tamarillo fruit on a non-structural four-point scale from 0–3, with 0 = not detectable, 1 = weak, 2 = moderate, 3 = strong [20]. Descriptors used were determined in preliminary sensory experiments. Each descriptor used was defined on the basis of the odor of a reference compound dissolved in water at a concentration of 100 times above the respective threshold value (Table 1). Reference odorants were: ethyl butanoate (fruity), (*Z*)-3-hexenal (herbal-green), 1,8-cineole (fresh-mint), ethyl-3-hydroxybutyrate (rancid), and terpinen-4-ol (earthy). Before the analysis, panelists were trained in the recognition of descriptors, and also in the handling of intensity scales. Finally, the data were analyzed by variance and regression analysis and average values were compared using Tukey’s test with a probability *p* ≤ 0.05. These results were plotted in a spider web diagram. All samples were evaluated in two replicate sessions.

For recombination experiment, appropriate amounts (20–100 μL) of ethanolic stock solutions of the quantified odorants were mixed and made up to 1 L with water to yield the same concentrations as determined in yellow tamarillo fruit. Final ethanol concentration was kept below 1 g/L, that is, below the odor threshold of ethanol. The overall aroma profile of this model mixture (recombinate 1) was determined in the same way as described above for the fresh fruit. Based on the analyses of the panel, a second recombinate was prepared in the same manner described above, but with a 4.2 times less of (*Z*)-3-hexenal, and also sensory-compared with the fruit puree.

*Taste*. An experienced sensory panel consisting of eight judges (ages 24–40 years old) from Disaromas S.A., evaluated the taste attributes of different test solutions prepared from fresh fruit puree, fractions of yellow tamarillo, and rosmarinic acid. Prior to analysis, all panelists were trained with reference solutions for bitterness (caffeine), salty (NaCl), sourness (citric acid), and umami (sodium glutamate). Panelists were instructed to rate each attribute in each sample. Then, once the fractions of interest were selected, they underwent further separation and rated the intensities of taste attributes of interest on a line scale (data not shown). All fractions from food samples for taste analysis, prior to sensory testing, were liberated from solvent by rotary evaporation and were subsequently freeze-dried twice. The samples for sensory analyses were prepared in the same concentration found in the fruit (Table 1), following the ethics protocol approved by Science Faculty of Universidad Nacional de Colombia-Sede Bogotá (Acta 05, 4 May 2015).

The bitter taste threshold of rosmarinic acid is here reported for the first time in the literature, and was determined by the trained panel of Disaromas S.A., following the ASTM procedure. This method of limits with an ascending concentration series, use three-alternative forced-choice (3AFC) judgments at each concentration step [21]. It consists of a series of 3AFC presentations, each containing one rosmarinic acid sample and two blank samples. In order to allow a testing series of five 3AFC presentations, five discrete concentration (39–293 ppm) steps of the rosmarinic acid with a constant dilution factor of 1.5 per step throughout the scale were made, in duplicate. In addition, two blank presentations in which filtered water was used as solvent were also evaluated. For each 3AFC presentation, the judge is required to discriminate the sample that is different from the other two. The threshold estimation was based on the stopping rule (last reversal) and the group best-estimate thresholds (BETs) was the geometric average of individual BETs. The taste odor threshold was expressed in concentration units (mg/L).

### 3.12. Statistical Analysis

For principal component analysis (PCA), RWizard (version 7.0) was used.

## 4. Conclusions

The sensomics approach allowed the identification of the key aroma compounds and one bitter-active compound in yellow tamarillo fruit. The key aroma compounds in this fruit are C_6_-aliphatic compounds, such as (*Z*)-3-hexenal and hexanal; esters, such as ethyl butanoate; and terpenols, such as 1,8-cineole, exhibiting green, fruity, and minty odor notes, respectively. This is the first time that rosmarinic acid has been reported as a bitter compound as well as its taste threshold value. These results are expected to improve the sensory quality of added-value products from yellow tamarillo fruit.

## Figures and Tables

**Table 1:** Odor-active volatile compounds detected in SAFE extract of yellow tamarillo (*Solanum betaceum* Cav.) fruit.

No. ^a^	Compound ^b^	Odour Description ^c^	RI ^d^ FFAP HP-5		FD ^e^	Conc ± SD (% CV)	Odour Threshold	OAV ^h^
						(μg/kg Fresh Fruit) ^f^	(μg/kg Water) ^g^	
1	Methyl butanoate	Sweet, fruity	962	706	16	1758.5 ± 5.6 (0.3)	60	29
2	Ethyl butanoate	Fruity, citric	1062	784	512	776.2 ± 2.3 (0.3)	0.76	1021
3	Hexanal	Herbal-green	1091	776	16	565.4 ± 3.9 (0.7)	0.25	2262
4	(*Z*)-3-Hexenal	Green	1153	801	1024	4765.3 ± 13.0 (0.3)	0.25	19,061
5	1,8-Cineole	fresh, minty	1231	1022	512	847.0 ± 8.9 (1.1)	12	71
6	Hexanol	Fruity	1350	854	16	222.6 ± 40.0 (18)	2500	<1
7	4-Hydroxy-4-methyl-2-pentanone	Fruity	1373	816	64	2479.2 ± 10.2 (0.4)	280	9
8	(*Z*)-3-Hexenol	Green	1404	839	2	1655.1 ± 17.0 (1)	70	24
9	Ethyl 3-hydroxy-butanoate	Citric	1519	959	8	775.3 ± 47.0 (6.1)	230	3
10	2,3-Butanediol	Rancid	1539	777	256	81.5 ± 23.8 (29.4)	150	<1
11	Terpinen-4-ol	Cooked, earthy	1601	1164	512	548.8 ± 22.0 (4)	330	2

^a^ Odorants were numbered according to their retention time on the FFAP column; ^b^ odorants were identified by comparing their retention indexes on the FFAP column, their mass spectra, and their odor notes with respective data of reference compounds; ^c^ odor note as perceived at the sniffing port during GC-O analysis; ^d^ retention index; ^e^ flavor dilution factor; ^f^ all data are the mean of three measurements ± standard deviation. In all of the cases, correlation coefficient was higher than 0.975; ^g^ Leffingwell [11]; ^h^ OAV = Odor Activity Value, concentration divided by odor threshold.

## References

[1] Schieberle P., Hofmann T., Jelen H. (2011). Mapping the combinatorial code of food flavors by means of the molecular sensory science concept. Food Flavors—Chemical, Sensory and Technological Properties.

[2] Schieberle P., Hofmann T., Taylor A.J., Mottram D.S. (2014). Elucidation of the chemosensory code of food by means of a SENSOMICS approach. Flavour Science, Proceedings of the XIV Weurman Flavour Research Symposium.

[3] Suess B., Brockhoff A., Meyerhof W., Hofmann T. (2016). The odorant (*R*)-citronellal attenuates caffeine bitterness by inhibiting the bitter receptors TAS2R43 and TAS2R46. J. Agric. Food Chem..

[4] Vera De Rosso V., Mercadante A.Z. (2007). HPLC-PDA-MS/MS of anthocyanins and carotenoids from *Dovyalis* and T*amarillo* fruits. J. Agric. Food Chem..

[5] Vasco C., Ruales J., Kamal-Eldin A. (2008). Total phenolic compounds and antioxidant capacities of major fruits from Ecuador. Food Chem..

[6] Vasco C., Avila J., Ruales J., Svanberg U., Kamal-Eldin A. (2009). Physical and chemical characteristics of golden-yellow and purple-red varieties of tamarillo fruit (*Solanum betaceum* Cav.). Int. J. Food Nutr..

[7] Espin S., Gonzalez-Manzano S., Taco V., Poveda C., Ayuda-Durán B., Gonzalez-Paramas A.M., Santos-Buelga C. (2016). Phenolic composition and antioxidant capacity of yellow and purple-red *Ecuadorian cultivars* of tree tomato (*Solanum betaceum* Cav.). Food Chem..

[8] Torrado A., Suárez M., Duque C., Krajewski D., Neugebauer W., Schreier P. (1995). Volatile constituents from tamaillo (*Cyphomandra betaceae* Sendtn.) fruit. Flavour Fragr. J..

[9] Durant A.A., Rodríguez C., Santana A.I., Herrero C., Rodríguez J.C., Gupta M.P. (2013). Analysis of volatile compounds from *Solanum betaceum* Cav. fruits from Panama by head-space micro extraction. Rec. Nat. Prod..

[10] Forero D.P., Orrego C.E., Peterson D.G., Osorio C. (2015). Chemical and sensory comparison of fresh and dried lulo (*Solanum quitoense* Lam.) fruit aroma. Food Chem..

[11] Leffingwell & Associates (2008). Odor Detection Thresholds and References. http://www.leffingwell.com/odorthre.htm.

[12] Steinhaus M., Sinuco D., Polster J., Osorio C., Schieberle P. (2009). Characterization of the key aroma compounds in pink guava (*Psidium guajava* L.) by means of aroma re-engineering experiments and omission tests. J. Agric. Food Chem..

[13] Akşit H., Çelik S.M., Özkan Ş., Erenler R., Demirtaş İ., Telci İ., Elmastaş M. (2014). Complete isolation and characterization of polar portion of *Mentha dumetorum* water extract. Rec. Nat. Prod..

[14] Zadra M., Piana M., Faccim de Brum T., Boligon A.A., Borba de Freitas R., Mansur Machado M., Terra Stefanello S., Antunes Soares F.A., Linde Athayde M. (2012). Antioxidant activity and phytochemical composition of the leaves of *Solanum guaraniticum* A. St.-Hil. Molecules.

[15] Engel W., Bahr W., Schieberle P. (1999). Solvent assisted flavour evaporation-a new and versatile technique for the careful and direct isolation of aroma compounds from complex food matrices. Eur. Food Res. Technol..

[16] Schieberle P., Goankar A. (1995). Recent developments in methods for analysis of flavor compounds and their precursors. Characterization of Food: Emerging Methods.

[17] Grosch W. (1994). Determination of potent odorants in foods by Aroma Extract Dilution Analysis (AEDA) and calculation of odour activity values (OAVs). Flavour Fragr. J..

[18] IOFI Working Group on Methods of Analysis (2011). Guidelines for the quantitative gas chromatography of volatile flavouring substances, from the Working Group on Methods of Analysis of the International Organization of the Flavor Industry (IOFI). Flavour Fragr. J..

[19] Isaza J.H., Ito H., Yoshida T. (2004). Oligomeric hydrolyzable tannins from *Monochaetum multiflorum*. Phytochemistry.

[20] Lawless H.T., Heymann H. (2010). Sensory Evaluation of Foods: Principles and Practices.

[21] American Society of Testing and Materials (ASTM) (2005). American Society of Testing and Materials (ASTM). Standard practice for determining odor and taste thresholds by a forced-choice ascending concentration series of methods of limits, E679–04. Annual Book of Standards, American Society of Testing and Materials.

